# CIPROFLOXACIN-INDUCED NEUROTOXICITY — A RARE PRESENTATION

**DOI:** 10.51894/001c.123091

**Published:** 2024-08-30

**Authors:** Sumit Gami

**Affiliations:** 1 Internal Medicine Detroit Medical Center Sinai-Grace Hospital

## 80

### INTRODUCTION

Ciprofloxacin (CPX) is a fluoroquinolone antibiotic used for treating respiratory, urinary tract, gastrointestinal, and abdominal infections. Drowsiness, headaches, and dizziness are the most commonly reported symptoms. After discontinuation of the drug therapy, these usually disappear. In therapeutic doses, only a few studies have been conducted regarding the neurological adverse effects of this drug. Accordingly, we report that CNS events have included agitation, delirium, confusion, and organic psychosis in the present study.

### CASE DESCRIPTION

This is a 44-year-old alcoholic male presented with a complaint of breakthrough seizure and post-ictal confusion. He had acute on chronic alcoholic liver failure. CT abdomen and pelvis showed cirrhotic liver and moderate ascites, and underwent paracentesis twice with no evidence of spontaneous bacterial peritonitis. He had spiked a fever of 38.8°C, tachycardia, WBC 18k, Prothrombin time of 15, Albumin 2.1, and the initial concern was SBP versus pneumonia. He was started on antibiotics. Chest X-ray reported bibasal consolidation. Given negative culture for paracentesis fluid, blood, and sputum, antibiotics were de-escalated. He was treated with lactulose and rifaximin for hepatic encephalopathy prevention and ciprofloxacin for SBP prophylaxis. He returned to his baseline mentation, but four days after starting ciprofloxacin, he had a deterioration in mentation. He was agitated and delirious, disoriented, and had behavioral impairment too. He had visual hallucinations but denies tactile, or auditory hallucinations. He had upper limb ataxia but no nystagmus. He had no ongoing infections, and his Vit B12, folate, Syphilis EIA, urinalysis, UDS, and Ammonia were within normal limits. He was held ciprofloxacin for SBP prophylaxis for 48 hours to check if delirium improved and he improved drastically in 48 hours. He was managed conservatively with a sitter and frequent reorientation, MRI, and EEG to rule out subclinical seizures.

### DISCUSSION

Fluoroquinolones are also associated with CNS disturbances. GABA-A receptor inhibition and excitatory NMDA receptor activation may play a role in fluoroquinolone-mediated CNS toxicity. Studies have concluded that even repeated pharmacological dosages of ciprofloxacin cause neurological damage. In order to prevent potentially serious adverse reactions, dose reductions should be considered.

